# Evolution of minimally invasive cholecystectomy: a narrative review

**DOI:** 10.1186/s12893-024-02659-x

**Published:** 2024-11-29

**Authors:** Changjin Nam, Jun Suh Lee, Ji Su Kim, Tae Yoon Lee, Young Chul Yoon

**Affiliations:** 1 Kyungpook National University Medical College, Daegu, Republic of Korea; 2 Department of Surgery, Incheon St. Mary’s Hospital, College of Medicine, The Catholic University of Korea, Seoul, Republic of Korea

**Keywords:** Gallbladder disease, Cholecystectomy, Surgical technique, Minimally invasive surgery, Laparoscopy

## Abstract

**Background:**

Laparoscopic cholecystectomy, being a prevalent abdominal surgical procedure, has transitioned through various innovative stages aimed at reducing the procedure's invasiveness. These stages encompass Single-Incision Laparoscopic Cholecystectomy (SILC), Mini Laparoscopic Cholecystectomy (MLC), Natural Orifice Transluminal Endoscopic Surgery (NOTES), and Robotic-Assisted Laparoscopic Cholecystectomy (RALC). The purpose of this review is to trace the evolution of minimally invasive cholecystectomy techniques, assess their status, and identify emerging trends and challenges in the field.

**Method:**

An extensive review was performed to explore the evolution and characteristics of SILC, MLC, NOTES, and RALC. The approach involved an in-depth examination of literature available on PubMed, coupled with a critical assessment of surgical outcomes, associated complications, and technical hurdles posed by these methods.

**Results:**

SILC, despite its potential for reduced scarring, exhibits an elevated risk of bile duct damage and incisional hernia occurrences. MLC, mirroring the standard technique closely, presents minor benefits without amplifying postoperative complications, hence, positing itself as a feasible choice for routine elective cholecystectomy. NOTES, although still facing technical challenges, the hybrid transvaginal procedure is gaining clinical interest. RALC, heralded for its augmented precision and dexterity, emerges as a potential future avenue, although necessitating further exploration to ascertain its efficacy and safety.

**Conclusion:**

The progression of laparoscopic cholecystectomy methodologies embodies the surgical society's aspiration to minimize invasiveness whilst enhancing patient outcomes. This review endeavors to offer a structured discourse on SILC, MLC, NOTES, and RALC, aspiring to aid the ongoing deliberation on the judicious selection of surgical techniques in clinical practice.

**Supplementary Information:**

The online version contains supplementary material available at 10.1186/s12893-024-02659-x.

## Introduction

Laparoscopic cholecystectomy (LC) has become the preferred method for gallbladder removal, offering benefits such as less postoperative pain, faster recovery, shorter hospital stays, better cosmetic outcomes and increased patient satisfaction [1–3].The shift towards LC was not initially driven by clinical trials but by a consensus on its benefits within the medical community. By the time randomized trials were conducted, LC's advantages were already well recognized, helping it become the gold standard for treating symptomatic cholelithiasis [4].

**Fig. 1:**
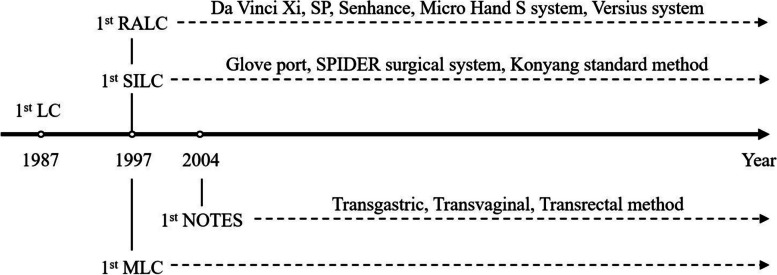
Timetable for the evolution of the LC modality

**Fig. 2:**
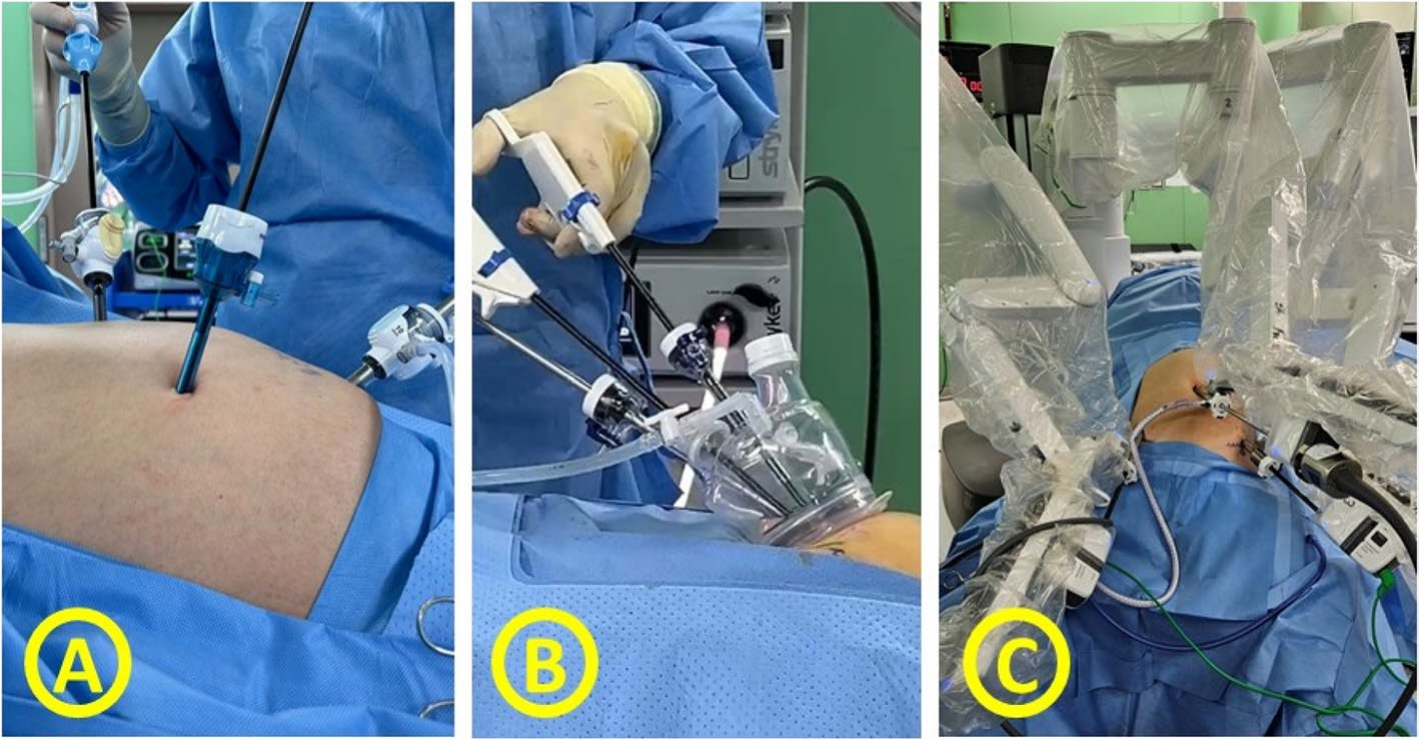
Three representative methods of minimally invasive cholecystectomy. **A** Conventional laparoscopic cholecystectomy, using 3 ports. **B** Single incision laparoscopic cholecystectomy, using a single incision through the umbilicus. **C** Robot assisted laparoscopic cholecystectomy. The robotic surgical system is docked to the patient’s abdomen

Currently, many different methods of minimally invasive cholecystectomy are being performed. Fig. 1 shows a brief timetable of the evolution of surgical technique for minimally invasive cholecystectomy. Conventional Laparoscopic Cholecystectomy (CLC), using four ports, has evolved to reduce invasiveness. Mini Laparoscopic Cholecystectomy (MLC) utilizes smaller incisions for the same procedure [5], while Needlescopic surgery, a subset of MLC, uses ≤ 3 mm diameter instruments [6]. Single Incision Laparoscopic Cholecystectomy (SILC) further minimizes incisions by using only one incision [7, 8]. Natural Orifice Transluminal Endoscopic Surgery (NOTES) eliminates external incisions, accessing the target organ via natural orifices [9]. Lastly, Robotic-Assisted Laparoscopic Cholecystectomy (RALC) introduces robotic technology for enhanced surgical precision and control [10], marking the latest advancement in minimally invasive gallbladder surgery. Fig. 2 shows three representative methods of minimally invasive cholecystectomy, CLC, SILC, and RALC.

This review aims to trace the evolution of minimally invasive cholecystectomy techniques, assess their status, and identify emerging trends and challenges. We focus on advancements in single-incision laparoscopic cholecystectomy (SILC), mini-laparoscopic cholecystectomy (MLC), natural orifice transluminal endoscopic surgery (NOTES), and robotic-assisted laparoscopic cholecystectomy (RALC) for treating gallbladder diseases. While many surgeons are familiar with these procedures, our target audience extends beyond experienced practitioners. We seek to serve a diverse readership, including general surgeons looking for the latest developments, surgical trainees requiring a thorough overview, researchers interested in comparative outcomes, and medical students pursuing an in-depth understanding of surgical advancements.

## Methods

This narrative review aims to provide a comprehensive overview of the evolution and current status of minimally invasive cholecystectomy techniques. We employed a systematic approach to literature search and selection, while maintaining the flexibility inherent to narrative reviews to capture the breadth of developments in this rapidly evolving field.

### Search strategy

We conducted a comprehensive literature search using PubMed, Embase, and Cochrane Library. The search included articles published up to August 2024. We used a combination of Medical Subject Headings (MeSH) terms and free-text keywords, including but not limited to: "laparoscopic cholecystectomy," "single-incision laparoscopic cholecystectomy," "mini-laparoscopic cholecystectomy," "natural orifice transluminal endoscopic surgery", "cholecystectomy," and "robotic-assisted cholecystectomy."

### Inclusion criteria

We included articles that met the following criteria: original research articles, systematic reviews, meta-analyses, and notable case series; focus on minimally invasive cholecystectomy techniques; published in peer-reviewed journals; and full text available in English.

### Exclusion criteria

We excluded articles that focused solely on open cholecystectomy techniques, were published as abstracts only, letters, or commentaries without original data, or described techniques in non-human subjects.

### Article selection process

Two reviewers independently screened titles and abstracts of retrieved articles. Full texts of potentially eligible articles were then assessed for inclusion. Disagreements were resolved through discussion with a third reviewer. We did not apply strict quality assessment criteria, as our goal was to capture the full spectrum of developments in minimally invasive cholecystectomy. However, we critically appraised each included study, considering factors such as study design, sample size, and potential biases.

### Data extraction and synthesis

From each included article, we extracted data on study characteristics (author, year, country, study design), patient demographics, surgical technique details, outcomes, and authors' conclusions and recommendations. Given the narrative nature of this review, we synthesized the extracted data qualitatively, organizing our findings thematically to trace the evolution of minimally invasive cholecystectomy techniques and highlight current trends and challenges.

## Single-Incision Laparoscopic Cholecystectomy (SILC)

### Introduction

#### Definition of SILC

Compared to open cholecystectomy, CLC offers benefits such as reduced pain and lower risks of wound infection or hernia due to less abdominal trauma. Building on this, SILC was developed in the late 1990s [7], introducing a technique where multiple instruments are inserted through a single navel incision, promoting it as a 'scarless surgery'.

#### Abbreviation of the existing literature

Table 1 summarizes the advantages and disadvantages of SILC over CLC (Additional file).

In assessing the efficacy of SILC versus CLC, findings from 9 Systematic Reviews (SRs) were analyzed [11–19]. The synthesis of these studies revealed that SILC offers better cosmetic outcomes than CLC but falls short in terms of operation duration, need for additional ports, incisional hernia rates, and costs. No significant differences were noted between SILC and CLC regarding conversion rates, overall complications, bile duct injury (BDI) rates, hospital stay length, and quality of life (QoL). Pain outcomes were mixed, with six SRs finding SILC either better or comparable to CLC, while one SR favored CLC for pain management.

### Surgical method

#### Basic surgical procedures

SILC aims to reduce port usage, typically employing a single incision in the umbilical area for the camera and the surgical instruments. However, the method faces challenges such as limited retraction and triangulation due to the close arrangement of instruments, which complicates the exposure of Calot's triangle and can lead to instrument collisions during precise dissections [1].

#### Konyang standard method (KSM)

The Konyang Standard Method (KSM), devised at Konyang University in 2010, addresses the limitations of traditional SILC, particularly for severe cholecystitis or gallstones. KSM utilizes three trocars (5, 10, and 12 mm) and flexible instruments to improve the surgical field and minimize complications, demonstrating enhanced outcomes in surgical time, length of hospital stay (LOH), and bleeding volume [20].

### Ports and instruments

#### Different types of ports used (SILS™ port vs Glove port)

SILC, which involves using a single port for laparoscopic cholecystectomy, includes various port types like the SILS™ port and the glove port. The SILS™ port is an elastic, hourglass-shaped device with one insufflation and three trocar openings for 5-12 mm trocars [21, 22]. Alternatively, the glove port utilizes a sterilized surgical glove with trocars inserted into the glove's fingers, offering a cost-effective option for single-incision surgery, particularly in resource-limited settings [22, 23].

#### Instrument designs

Research has shown that using curved instruments is more challenging than using linear instruments. This increases the difficulty due to the need for auxiliary measures and prolongs surgical time [24].

### Complications

Research suggests SILC is a safe and viable option with a low complication rate [25]. The available systematic reviews indicate that SILC's overall complication and Bile Duct Injury (BDI) rates are comparable to CLC, with a noted increase in incisional hernias post-SILC [11, 13, 17–19].

#### Bile Duct Injury [BDI]

BDI is a severe complication of cholecystectomy, sometimes necessitating lifelong management. The Critical View of Safety (CVS) technique is widely adopted to minimize BDI risk during cholecystectomy by ensuring clear visibility of key anatomical structures [26]. However, the unique challenges of SILC, such as limited visibility and instrument crowding, can hinder achieving CVS [16]. To mitigate this, Magnetic Resonance Cholangio-Pancreatography (MRCP) has has been used to better delineate biliary anatomy pre-surgery [27]. An alternative method was reported, using intravenous injection of indocyanine green and direct fluorescent cholangiography [28]. However, due to the abundant adipose tissue, the effectiveness was compromised in obese patients.

#### Incisional hernia

Laparoscopic surgery, known for its minimal incisions and reduced hernia risk, faces a challenge with SILC due to its single larger incision potentially increasing hernia risk [15, 19]. Studies on hernia rates post-SILC versus CLC have shown mixed results. Two retrospective cohort studies observed higher hernia rates following SILC compared to CLC [29, 30], while another study reported no significant difference [31]. Additionally, a 2021 meta-analysis of 89 studies indicated a higher hernia occurrence after SILC at 6- and 12-months post-operation [32]. Suggestions to mitigate hernia risk include preferring SILC for younger, non-obese patients [33].

These varied findings on SILC complications like BDI and incisional hernia underscore the necessity for standardized SILC techniques and better complication management strategies.

### QoL

#### Cosmetic satisfaction

According to the majority of the 9 SRs previously analyzed, SILC has significant benefits over CLC in terms of aesthetics and body image [12–15, 17, 19]. This benefit arises from the fact that SILC requires fewer incisions, making it cosmetically more appealing.

#### Comprehensive QoL

Furthermore, 6 of the SRs suggest that SILC either has an advantage over CLC in terms of postoperative pain [12, 13, 15] or shows no significant difference [17–19]. However, one study indicated that SILC might be at a disadvantage compared to CLC in terms of pain [34]. The influence of SILC on postoperative pain varies across studies, implying the need for further research in this domain. When comparing the overall QoL, there appears to be no significant difference between SILC and CLC [13].

#### Cost

It has been reported that SILC is significantly more expensive than CLC [17]. This higher cost is attributed to the specially designed single-port systems and instruments for SILC. This cost factor could pose a barrier to the further integration of SILC.

### Additional comments

#### Indications for SILC

The criteria for SILC application vary, reflecting ongoing debates. A Japanese study with 232 patients outlined SILC's effective indications as preventive surgery, age under 60, and BMI under 30 kg/m2, while contraindications included a thick gallbladder wall, prior abdominal surgery, bile duct stones, and severe comorbidities [35]. Conversely, a larger involving 6,497 patients suggested that technological advancements have broadened SILC's applicability, even for patients with acute cholecystitis [36].

#### Surgeon factor

The surgeon's expertise and level of training are pivotal for the successful performance of SILC, which is technically more demanding and stressful than CLC. Studies have shown that SILC places greater physical and mental strain on surgeons, as evidenced by higher salivary cortisol levels and increased heart rate during SILC procedures compared to CLC [37]. This may be because single incision surgery requires an ergonomically uncomfortable position, resulting in greater muscular activity [38].

The surgical training continuum for SILC involves a progression from competency to proficiency. Competency refers to the basic ability to perform the procedure safely and effectively, typically involving completion within an acceptable timeframe and with minimal complications. Proficiency, on the other hand, goes beyond this basic level, indicating a high degree of skill, efficiency, and consistency. This progression highlights the importance of continued training and experience in mastering the technically demanding SILC procedure.

### Conclusion

SILC represents a significant advancement in laparoscopic surgery, enhancing patient satisfaction through better cosmetic outcomes, positive body image perceptions, and quicker recovery times. Ongoing debates about SILC's safety and effectiveness highlight concerns over complications such as BDI and incisional hernia. Emphasizing the need for precise bile duct identification can help reduce these risks, with standardized techniques expected to improve safety. While SILC may incur higher costs compared to CLC, the evolving criteria for its application and the importance of surgeon expertise are key areas for ongoing study and development.

## Mini-laparoscopic cholecystectomy(MLC)

### Introduction

#### Definition of MLC

The introduction of MLC in the late 1990s marked a significant advancement in laparoscopic surgery, aiming to minimize incision sizes further by using ports smaller than 3 mm [5, 6, 39, 40]. Despite the insertion points for MLC being similar to those of CLC, MLC demands greater surgical skill due to the reduced size of the ports. These smaller ports lead to decreased instrument rigidity and maneuverability [41]. Nonetheless, MLC offers the benefit of being less invasive and maintains the critical aspect of triangulation, which is often compromised in SILC.

#### Abbreviation of the existing literature

Tables 1 and 2 summarizes the advantages of disadvantages of MLC over CLC and SILC.

**Table 1:**
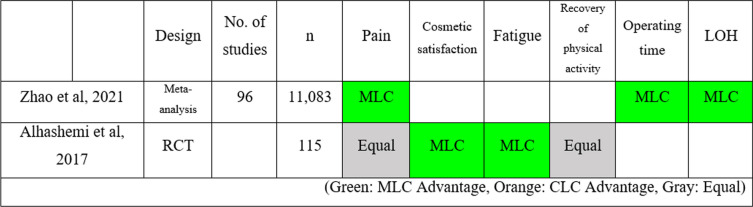
Advantages and disadvantages of MLC over other methods of cholecystectomy. **A** A comparison of MLC and CLC

(Green: MLC Advantage, Orange: CLC Advantage, Gray: Equal)

Comparing MLC with CLC, previous studies [42, 43] have shown MLC's superiority in reducing pain, enhancing cosmesis, lessening fatigue, shortening operating times, and decreasing the LOH. However, both techniques were similar in the postoperative recovery.

**Table 2:**
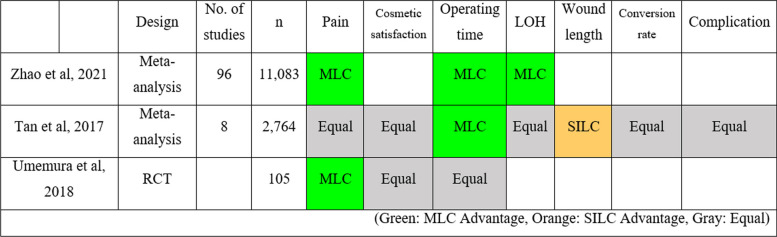
Advantages and disadvantages of MLC over other methods of cholecystectomy. **B **A comparison of MLC and SILC

(Green: MLC Advantage, Orange: SILC Advantage, Gray: Equal)

In comparing MLC to SILC, analyses from two meta-analyses and one RCT [43–45] indicated that MLC generally surpasses SILC in reducing pain, operating time, and LOH. There were no notable differences in cosmetic satisfaction, conversion rates, and complications between MLC and SILC, although SILC had a shorter wound length advantage.

### Complications

From a previous meta-analysis, no significant difference was found in the occurrence of complications between MLC and SILC [44]. However, due to the use of smaller ports in MLC, there might be a suboptimal video quality. This can increase the risk of gallbladder wall perforation and spillage of gallbladder contents [44].

### Conclusion

MLC is recognized for its potential to lessen post-operative pain and offer superior cosmetic results due to smaller incisions. Despite these benefits, MLC introduces challenges such as reduced instrument stability and visual limitations. Comparative studies between MLC and CLC have generally shown no significant difference in complication rates. However, the smaller ports used in MLC may heighten the risk of complications like gallbladder wall damage and bile spillage, highlighting the need for surgical skill and experience.

## Natural orifice transluminal endoscopic surgery(NOTES) cholecystectomy

### Introduction

#### Definition of NOTES

Introduced in 2004 [46], NOTES represents a surgical technique where endoscopic and surgical instruments are introduced through natural orifices such as oral, vaginal, or rectal to remove the gallbladder. NOTES cholecystectomy is generally considered more minimally invasive than CLC.

#### Abbreviation of the existing literature

Table 3 summarizes the advantages and disadvantages of NOTES over CLC.

**Table 3:**
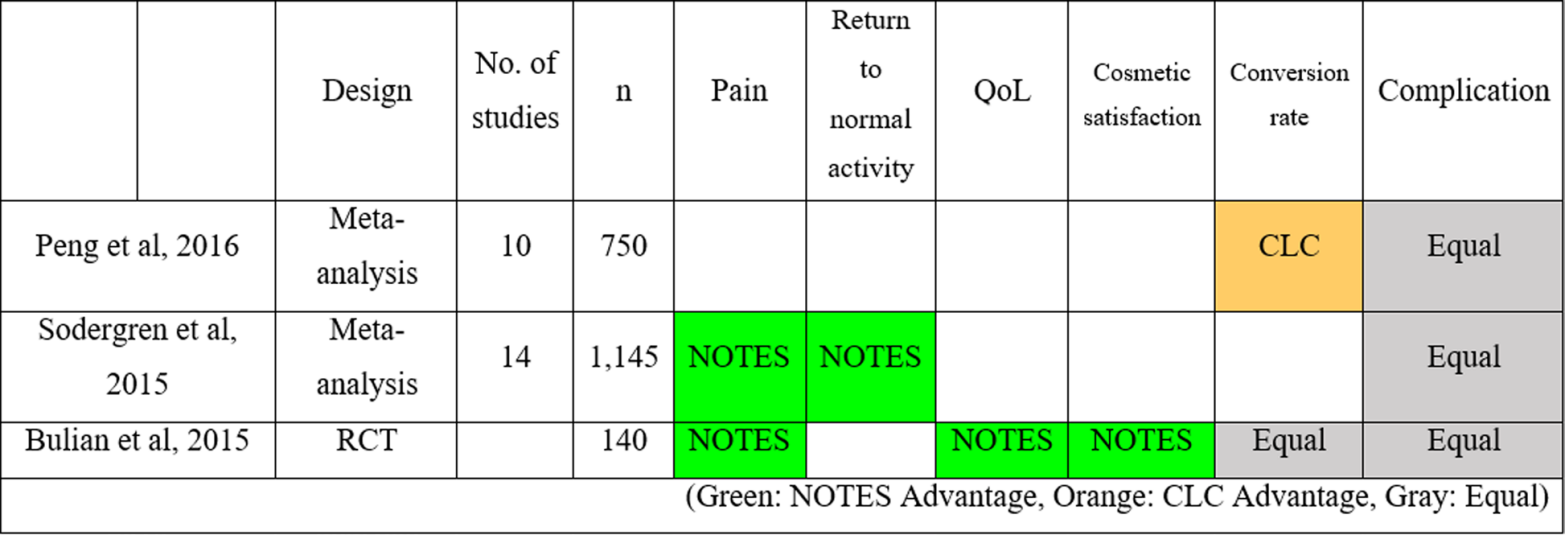
Advantages and disadvantages of SILC over CLC

Green: SILC Advantage, Orange: CLC Advantage, Gray: Equal

To compare the efficacy of NOTES cholecystectomy with CLC, two meta-analyses and one RCT were referenced [47–49]. According to these studies, NOTES cholecystectomy mostly outperformed CLC in terms of return to normal activity, pain, QoL, and cosmetic satisfaction. However, the complication rates between the two methods showed no significant difference. In terms of conversion rate, CLC was superior to NOTES. An important limitation of this comparison between NOTES and CLC is that the different types of NOTES are all assumed to be a single surgical technique, for the purpose of comparison. The details and nuances of each technique are discussed in the following respective subsections.

### Surgical method

NOTES cholecystectomy can be categorized into pure and hybrid based on the instruments used and the approach employed. A Pure NOTES cholecystectomy is conducted solely with an endoscope, while a Hybrid NOTES cholecystectomy combines both endoscopy and laparoscopy. Furthermore, NOTES can be classified based on the route of entry to the abdominal cavity, with the principal methods for cholecystectomy being trans-gastric, -vaginal, and -rectal [50].

#### Transgastric NOTES(TG-NOTES) chlecystectomy

Most TG-NOTES cholecystectomy procedures performed to date required at least one transabdominal port to ensure safety by exposing Calot’s triangle. Gastrotomy was carried out using an endoscopic method under direct visualization from laparoscopy, with laparoscopic suturing. Gallbladders exceeding 2 cm were typically retrieved through an abdominal port as esophageal extraction proved challenging. A unique challenge of TG-NOTES cholecystectomy is that endoscopic dissection is only feasible in a retroverted position [50, 51].

#### Transvaginal NOTES(TV-NOTES) cholecystectomy

The number of TV-NOTES procedures has steadily increased since its inception with 9 cases in 2007 [52, 53]. Much like TG, Hybrid procedures outnumbered Pure ones. The cystic duct ligation employed surgical clips because endoclips weren’t designed to fully occlude tubular structures [50].

#### Transrectal NOTES (TR-NOTES) cholecystectomy

TR-NOTES, primarily explored for lower rectal cancer treatments due to concerns over fecal contamination, has seen limited use compared to TG- and TV-NOTES. Nonetheless, its application in cholecystectomy persists. In 2016, advancements in rectoscope and trocar technology facilitated the development of a device enhancing transrectal trocar placement [54]. These innovations may pave the way for broader clinical adoption of TR-NOTES procedures in the future.

### Complications

Meta-analyses and an RCT comparing NOTES cholecystectomy with CLC found no significant differences in complication rates, indicating comparable safety profiles for both techniques [47–49]. Similarly, no significant difference in complications was observed between NOTES and MLC [55]. Concerns about sexual function changes after TV-NOTES cholecystectomy were addressed in a 2018 case series, which reported no adverse effects on sexual function in 47 patients, with high satisfaction rates regarding cosmetic outcomes [56]. These findings support the notion that NOTES cholecystectomy is as safe as traditional methods, suggesting a shift in perception towards the technique's safety and efficacy may be warranted.

### QoL

Two meta-analyses and one RCT comparing NOTES cholecystectomy with CLC, and another RCT comparing it with MLC, suggest that NOTES may be superior in various aspects such as overall quality of life, postoperative pain, recovery, and cosmetic satisfaction [48, 49, 55].

### Additional comments

#### Obese patients

A study involving 231 patients evaluated the impact of obesity on Hybrid TV-NOTES cholecystectomy outcomes, revealing that while procedural time, ASA classification, and the use of percutaneous trocars increased with higher BMI, there were no significant differences in complications, LOH, or fatalities. [57] Comparative research between TV-NOTES and CLC in obese patients highlighted TV-NOTES's benefits, such as reduced postoperative pain and shorter LOH, without significant differences in complication rates or operation duration. These findings suggest TV-NOTES offers short-term benefits for obese patients over CLC [58].

#### Patients with a History of Hysterectomy

Research on the usability and effects of TV-NOTES cholecystectomy in patients with a prior hysterectomy found that the procedure was more challenging in these patients, requiring additional laparoscopic ports and longer surgical durations. However, the rates of intra- and post-operative complications did not show significant differences when compared with those who hadn't undergone a hysterectomy [59].

### Conclusion

NOTES, a method using natural orifices for gallbladder surgery, has the benefits of less pain, quicker recovery, and better post-surgery life quality. NOTES cholecystectomy seemed to gain traction in its experimental phase, but interest in this procedure seems to be waning, due to concerns about potential bias in research and the necessity of its high technical demands. Another point to consider is whether NOTES's internal organ injury is preferable to abdominal wall injury from traditional methods like SILC or MLC.

## Robot-Assisted Laparoscopic Cholecystectomy (RALC)

### Introduction

#### Definition of RALC

RALC integrates robotic technology into laparoscopic cholecystectomy, enhancing surgical precision and efficiency. Since the introduction of robotic systems in the 1990s and the advent of the da Vinci Surgical System in the 2000s, RALC's popularity has significantly increased, with its usage rising from 0.02% to 3.2% of all cholecystectomies between 2008 and 2017 [60].

#### Abbreviation of the existing literature

A 2017 meta-analysis found no significant difference in LOH, conversion rates, or complications between RALC and CLC, although RALC generally required longer operating times [61]. Other studies, however, have indicated potential advantages of RALC, including lower conversion rates and reduced blood loss [62–64].

### Surgical method

RALC allows surgeons to operate with robotic arms controlled from a console, providing a 3D view of the operative field. This method offers several advantages over CLC, including enhanced dexterity through robotic wrist-like joint movements, superior three-dimensional visualization, and better image. These features are particularly beneficial for complex surgical tasks, such as managing advanced gallbladder cancer [65, 66]. However, the effectiveness of RALC can vary significantly depending on the robotic system, with the da Vinci Surgical System and Senhance being notable examples approved by the FDA [67].

#### Single-Incision Robotic Cholecystectomy(SIRC)

SIRC represents an advanced approach in minimally invasive surgery, leveraging robotics to enhance SILC. A comprehensive meta-analysis in 2023 highlighted SIRC's superiority in reducing post-operative complications such as BDI and LOH compared to SILC, MLC, and CLC [14]. The robotic systems' capabilities to mimic full joint movements, provide a 3D view, ensure image stability, and enhance instrument mobility significantly contribute to SIRC's lower learning curve and reduced surgeon stress, addressing limitations inherent in SILC [66].

SIRC has been deemed feasible and safe for a broader range of patients, including obese individuals, those with previous hysterectomies, and pediatric patients, as evidenced by various studies [68–70]. The da Vinci Xi and SP systems are predominantly used for SIRC, with the SP system, introduced in 2018, being specifically designed for single-port surgeries. Recent research confirms the SP system's safety and efficacy in adolescent patients [71], with minimal clinical differences observed between the Xi and SP models [72].

Emerging robotic platforms, including the Senhance system and the not-yet FDA-approved Micro Hand S and Versius systems, are broadening SIRC's applications. The Micro Hand S, a cost-effective Chinese innovation, and the UK-developed Versius, with its small, modular design, have both shown promise in enhancing surgical performance and safety, even in complex cases like obesity [73, 74].

### Additional comments

#### Financial aspect

The financial implications of adopting RALC present a significant challenge for hospitals, given the high costs associated with procurement and maintenance of robotic systems like the da Vinci. Initial costs for these systems can range between 0.6 to 2.5 million dollars, with annual maintenance fees of 100,000 to 170,000 dollars, leading to an additional 1,600 dollars per procedure. This increases the per-case cost to approximately 3,200 dollars when including the purchase price [75].

However, a study comparing RALC and CLC financials over two years at a single institution found no significant difference in net income between the two methods, suggesting that with strategic management, the financial gap can be narrowed [76]. The impending expiration of key patents in the robotic surgery field is expected to introduce more competition, potentially lowering costs and making RALC more accessible [75].

Despite the financial challenges, the demand for robotic surgery, driven by surgeon preference and market demand, encourages hospitals to invest in RALC. The ongoing development of policies and technologies aims to alleviate the financial burden, suggesting a future where RALC could become more widely adopted and financially viable.

### Conclusion

RALC offers surgeons advanced control, manipulation, and 3D visualization. Comparative studies highlight RALC's superiority over CLC in aspects such as lower conversion rates, reduced blood loss, shorter hospital stays, and fewer complications. RALC is also noted for its easier learning curve for beginners and its applicability to specific demographics like obese patients and children. In SIRC, the robotic system's advantages help overcome the limitations seen in SILC, making SIRC preferable for single-incision procedures.

The high cost of robotic systems poses a significant barrier to RALC's widespread adoption. However, studies indicating minimal differences in overall surgical costs between RALC and CLC, along with the potential for market competition following patent expirations, suggest financial viability.

## Conclusion

The evolution of surgical methods, especially in laparoscopic cholecystectomy, showcases the continuous endeavor to improve patient outcomes. However, the existing literature does not sufficiently support a comprehensive evaluation of the newly introduced minimally invasive techniques for LC. The techniques discussed, namely SILC, MLC, NOTES, and RALC, each come with their unique set of merits and challenges that require thorough exploration for beneficial clinical adoption. A summary of the advantages, limitations, and other notable aspects of each modality is provided in Table 4 (additional file).

This review reveals that none of the discussed innovative techniques have showcased clear advantages over CLC. Specifically, SILC, while aiming to minimize scarring, is linked with a heightened risk of BDI and a potential rise in incisional hernia incidences, rendering its current clinical recommendation premature. NOTES cholecystectomy seemed to gain traction in its experimental phase, but interest in this procedure seems to be waning, due to high technical demand and the need for internal organ injury. MLC, being nearly identical to the conventional technique, potentially offers minor advantages without increasing postoperative complications, thereby presenting itself as a suitable option for routine elective cholecystectomy.

Furthermore, the introduction of RALC adds a new dimension to this discussion. RALC, with its superior precision and control facilitated by robotic assistance, hints at a promising pathway forward. Nevertheless, its efficacy and safety necessitate rigorous scrutiny through well-designed clinical trials to ascertain its standing in the array of laparoscopic cholecystectomy techniques.

In summary, this review accentuates the need for a tailored approach to discern the most appropriate technique for each patient. Such deliberations are crucial not only for advancing surgical practice but also for ensuring patient safety and well-being. The ongoing effort to meticulously evaluate and refine surgical techniques is essential for transitioning towards an era of more efficacious and less invasive surgical interventions.

## Declaration of AI and AI-assisted technologies in the writing process

In the preparation of this manuscript, we utilized the AI language model ChatGPT (version 4o) developed by OpenAI.

### Method and questions posed

We requested assistance in phrasing complex medical concepts in clear, accessible language.

#### Extent of AI usage

We estimate that approximately 20% of the initial draft was generated with the assistance of ChatGPT. However, all technical content, data interpretation, and conclusions were directly input and overseen by the authors.

#### Final responsibility

While we utilized AI to assist in the writing process, the authors take full responsibility for the content and accuracy of this publication.

## Supplementary Information

Supplementary Material 1.

## Data Availability

No datasets were generated or analysed during the current study.
