# Correction: Evolution of minimally invasive cholecystectomy: a narrative review

**DOI:** 10.1186/s12893-024-02715-6

**Published:** 2024-12-17

**Authors:** Changjin Nam, Jun Suh Lee, Ji Su Kim, Tae Yoon Lee, Young Chul Yoon

**Affiliations:** 1 Kyungpook National University Medical College, Daegu, Republic of Korea; 2 Department of Surgery, Incheon St. Mary’s Hospital, College of Medicine, The Catholic University of Korea, Seoul, Republic of Korea

**Correction: BMC Surg 24, 378 (2024)**

**https://doi.org/10.1186/s12893-024-02659-x**

The original publication of this article [1] contained an incorrect grant number in Funding section. The incorrect and correct information is listed in this correction article. The original article has been updated.

Incorrect grant number in Funding

This work was supported by the National Research Foundation of Korea (NRF) grant funded by the Korea government (Ministry of Science and ICT, Grant number: 5-2024-A0154-00280).

Correct grant number in Funding:

This work was supported by the National Research Foundation of Korea (NRF) grant funded by the Korea government (Ministry of Science and ICT, Grant number: RS-2022-NR071874).
