# Supplementary material for: Evolution of minimally invasive cholecystectomy: a narrative review
Source: BMC Surg. 2024 Nov 29;24:378. doi: 10.1186/s12893-024-02659-x (PMC11606199; doi:10.1186/s12893-024-02659-x)
Supplement: Supplementary file 1 — Supplementary Material 1. [file 12893_2024_2659_MOESM1_ESM.docx]

Table 1. Advantages and disadvantages of SILC over CLC

|  |  |  |  |  |  |  |  |  | Complication | | |  |  |  |
| --- | --- | --- | --- | --- | --- | --- | --- | --- | --- | --- | --- | --- | --- | --- |
|  |  | No. of studies | n | Pain | Cosmetic satisfaction | Operation time | Additional port | Conversion  rate | Total | BDI | Hernia | Cost | QoL | LOH |
| Evers et al, 2017 | | 9 | 860 | SILC | SILC | CLC | CLC | Equal | CLC | Equal | CLC |  | Equal | Equal |
| Hauster et al, 2017 | | 37 | 3,051 | SILC | SILC | CLC | CLC | Equal |  |  | CLC |  |  |  |
| Lirici et al, 2016 | | 17 | 1,293 | Equal | SILC | CLC |  | Equal | Equal | Equal |  | CLC |  |  |
| Allemann et al, 2014 | | 11 | 898 |  |  |  |  |  | Equal | Equal |  |  |  |  |
| Milas et al, 2014 | | 30 | 2,411 | Equal | SILC | CLC | CLC |  | Equal |  | CLC |  |  |  |
| Arezzo et al, 2013 | | 12 | 996 | SILC | SILC | CLC |  |  |  |  |  |  |  |  |
| Markar et al, 2012 | | 7 | 375 | Equal |  | CLC |  |  | Equal |  |  |  |  | Equal |
| Hall et al, 2012 | | 49 | 2,336 | CLC | SILC |  |  |  |  |  |  |  |  |  |
| Joseph et al, 2012 | | 45 | 2,626 |  |  |  |  |  |  | CLC |  |  |  |  |
| Result | SILC |  |  | **3** | **6** | 0 | 0 | 0 | 0 | 0 | 0 | 0 | 0 | 0 |
|  | CLC |  |  | 1 | 0 | **6** | **3** | 0 | 1 | 1 | **3** | **1** | 0 | 0 |
|  | Equal |  |  | **3** | 0 | 0 | 0 | **3** | **4** | **3** | 0 | 0 | **1** | **2** |
| (Green: SILC Advantage, Orange: CLC Advantage, Gray: Equal) | | | | | | | | | | | | | | |

Table 4. A summary of the advantages, limitations, and other notable aspects of each modality of minimally invasive cholecystectomy

|  | **Benefits** | **Limitations** | **Other Notable Aspects** |
| --- | --- | --- | --- |
| **SILC** | * **Enhanced aesthetic outcomes** due to fewer incisions, leading to improved body image and cosmetic satisfaction. * Potential for **reduced postoperative pain** in some studies, with conflicting results suggesting variability among patient experiences. | * **Higher cost** compared to CLC due to specialized equipment. * Increased complexity of the procedure can lead to a **higher risk of bile duct injury and incisional hernias.** * Lack of standardized technique, leading to **heterogeneity in surgical approaches and outcomes.** | * **Surgeon expertise** plays a significant role in minimizing complications and improving outcomes. * Ongoing research and technological advancements may help overcome current limitations. |
| **MLC** | * **Reduced invasiveness** with smaller incisions, leading to less postoperative pain and improved cosmetic outcomes. * Maintains the advantages of CLC, including the **preservation of triangulation**, which is lost in SILC. | * Requires **higher surgical skill** due to reduced instrument rigidity and maneuverability. * Potential issues with **camera quality and lighting** due to smaller ports, which could increase the risk of complications such as gallbladder perforation. | * Can be **easily converted to CLC** if necessary, providing flexibility during surgery. * Innovations like the **Low-impact laparoscopic (LIL) cholecystectomy** approach show promise in further reducing invasiveness and postoperative pain. |
| **NOTES** | * **Eliminates abdominal incisions**, potentially reducing pain, and improving recovery times. * Offers an **enhanced quality of life post-surgery** due to minimal external scarring. | * **High technical demand and steep learning curve.** * Concerns about the **necessity and safety of internal organ injury** for access. | * The utility of NOTES over other minimally invasive techniques remains a topic of debate, with a need for more research to establish clear advantages. |
| **RALC** | * **Increased precision and control** through robotic assistance, potentially improving surgical outcomes. * **Lower or similar rates of conversion, blood loss, and readmissions** compared to CLC. | * **Significant initial investment and operating costs.** * Concern of complications such as bile duct injury. * Variability in outcomes depending on the specific robotic system used. | * Continuous technological advancements and system diversification are likely to expand the applicability and benefits of RALC. * The financial aspect remains a challenge, but with strategic planning and policy support, the adoption of RALC could become more feasible for more hospitals. |
